# A multiplex analysis of sepsis mediators during human septic shock: a preliminary study on myocardial depression and organ failures

**DOI:** 10.1186/s13613-019-0538-3

**Published:** 2019-06-04

**Authors:** Keyvan Razazi, Florence Boissier, Mathieu Surenaud, Alexandre Bedet, Aurélien Seemann, Guillaume Carteaux, Nicolas de Prost, Christian Brun-Buisson, Sophie Hue, Armand Mekontso Dessap

**Affiliations:** 1 AP-HP, Service de Réanimation Médicale, Hôpitaux universitaires Henri Mondor, DHU A-TVB, 94010 Créteil, France; 2 IMRB, GRC CARMAS, Faculté de Médecine de Créteil, Université Paris Est Créteil, 94010 Créteil, France; 3 Present Address: Réanimation médicale, CHU de Poitiers, Poitiers, France; 4 INSERM CIC 1402 (ALIVE Group), Université de Poitiers, Poitiers, France; 5 IMRB, Team 16, Faculté de Médecine, Université Paris Est Créteil, 94010 Créteil, France; 6 Vaccine Research Institute (VRI), 94010 Créteil, France; 7 AP-HP, Service d’immunologie, Hôpitaux universitaires Henri Mondor, 94010 Créteil, France

**Keywords:** Septic shock, Myocardial depression, Biomarkers, Mortality

## Abstract

**Background:**

The mechanisms of organ failure during sepsis are not fully understood. The hypothesis of circulating factors has been suggested to explain septic myocardial dysfunction. We explored the biological coherence of a large panel of sepsis mediators and their clinical relevance in septic myocardial dysfunction and organ failures during human septic shock.

**Methods:**

Plasma concentrations of 24 mediators were assessed on the first day of septic shock using a multi-analyte cytokine kit. Septic myocardial dysfunction and organ failures were assessed using left ventricle ejection fraction (LVEF) and the Sequential Organ Failure Assessment score, respectively.

**Results:**

Seventy-four patients with septic shock (and without immunosuppression or chronic heart failure) were prospectively included. Twenty-four patients (32%) had septic myocardial dysfunction (as defined by LVEF < 45%) and 30 (41%) died in ICU. Hierarchical clustering identified three main clusters of sepsis mediators, which were clinically meaningful. One cluster involved inflammatory cytokines of innate immunity, most of which were associated with septic myocardial dysfunction, organ failures and death; inflammatory cytokines associated with septic myocardial dysfunction had an additive effect. Another cluster involving adaptive immunity and repair (with IL-17/IFN pathway and VEGF) correlated tightly with a surrogate of early sepsis resolution (lactate clearance) and ICU survival.

**Conclusions:**

In this preliminary study, we identified a cluster of cytokines involved in innate inflammatory response associated with septic myocardial dysfunction and organ failures, whereas the IL-17/IFN pathway was associated with a faster sepsis resolution and a better survival.

**Electronic supplementary material:**

The online version of this article (10.1186/s13613-019-0538-3) contains supplementary material, which is available to authorized users.

## Introduction

Sepsis is a complication caused by the body’s overwhelming and life-threatening response to infection. This dysregulated host response to infection is mediated by several endogenous factors (involving inflammatory response and non-immunologic pathways) and may lead to tissue damage, organ failure and death. During sepsis, systemic activation of the innate immune system by microbes, microbial components and products of damaged tissue results in a severe and persistent inflammatory response characterized by an excessive release of inflammatory cytokines, known as the “cytokine storm.”

Circulatory failure is one of the hallmark alterations in sepsis and involves a variable combination of hypovolemia, vasoplegia and myocardial dysfunction [1]. Septic myocardial dysfunction was first described by Parker et al. 2]. Its mechanisms are not fully understood. Parrillo et al. [3] showed that sera obtained from patients with septic shock depressed rat myocardial cell contractility in vitro, and proposed the concept of a circulating myocardial depressant factor. Subsequent in vitro and experimental studies have proposed several cytokines as myocardial depressant factor candidates, including tumor necrosis factor alpha (TNF-α), interleukin-1 beta (IL-1β) and interleukin-6 (IL-6) [4–8]. These studies are limited by the variability of models and species used. Reports examining the association of circulating cytokine concentrations with septic myocardial dysfunction in the clinical setting are scarce [9]. The involvement of a large number of known [6–8] and emerging [e.g., soluble suppression of tumorigenicity-2 (sST2)] [10, 11] candidate sepsis mediators derived from experimental studies should be tested in human septic shock.

Besides septic myocardial dysfunction, the inflammatory response may be implicated in the pathophysiology of other organ failures and repair during septic shock, as well as in bacterial clearance. Sepsis mediators show a high degree of interaction. Their biological coherence (i.e., the consistency of different mediators involved in the same biological pathway), potential synergy and clinical significance have not been comprehensively assessed during human septic shock.

The aims of the present study were twofold: (1) first, to assess the biological coherence of a large panel of endogenous factors proposed to evaluate host response and damage during human septic shock and (2) second, to explore the role of these mediators over septic myocardial dysfunction, organ failures and outcome.

## Methods

### Patients

Patients who met septic shock criteria (as defined according to the ACCP/SCCM Consensus Conference [12]) were prospectively included at the medical ICU of Henri Mondor University Hospital (Creteil, France). Non-inclusion criteria were chronic heart failure [defined as a baseline left ventricle ejection fraction (LVEF) below 45%], severe valvulopathy or immunodepression (including human immunodeficiency virus infection, hematology malignancy, solid organ or bone marrow transplant, immunosuppressive therapy or chemotherapy). This study was approved by our institutional review board (CPP Ile de France IX), as a component of standard care, and informed consent was waived. Written and oral information about the study was given to the patients or families. Patient’s severity was evaluated by the McCabe and Jackson score for underlying diseases [13], the SAPS II score for acute illness at ICU admission [14] and the SOFA score for organ dysfunction at septic shock onset [15]. Norepinephrine was the first-choice vasopressor therapy (used to target a mean arterial pressure of 65 mmHg or more); dobutamine was added in the presence of decreased LVEF (< 45%) with ongoing signs of hypoperfusion despite adequate mean arterial pressure; epinephrine could be considered if the latter condition was not met. Surrogate of sepsis resolution was assessed with lactate clearance, calculated as the relative difference between lactate at septic shock onset and after 24 h of resuscitation [16]. Follow-up for the study was until hospital discharge.

### Echocardiography

To evaluate cardiac function, we repeated transthoracic or multi-plane transesophageal echocardiographies (when transthoracic route did not allow for accurate measurements because of poor acoustic windows) on the first and second days of septic shock. These echocardiographies were performed by trained operators (competence in advanced critical care echocardiography) using an iE33 system (Philips Ultrasound, Bothell, WA) with a standard procedure [17]. The four-chamber and two-chamber long-axis views were used to assess LVEF (computed from LV volumes using the biplane Simpson method when image quality was good or visually estimated when poor image quality did not allow sufficient identification of the endocardium). All measures were averaged over a minimum of three cardiac cycles (five to ten in case of non-sinus rhythm). On each assessment, LVEF was defined as depressed (< 45% or when an inotrope infusion was needed to achieve a value ≥ 45%) or preserved (≥ 45% with no inotrope infusion). Septic myocardial dysfunction was defined as the occurrence of a depressed LVEF on day 1 or on day 2.

### Sample preparation and cytokine measurement

On the first day of septic shock, we assessed plasma concentration of 24 putative sepsis mediators including inflammatory markers (IL-1α, IL-1β, IL1-RA, IL-6, IL-10, IL-12, IL-15, IL-17, IL-33, IFN-γ, TNF-α, CD40L, HSP70, sFAS, sFAS ligand, sST2, granzyme, TRAIL, PAI1 and VEGF), chemokines (IL-8, MCP1) and adhesion molecules (sVCAM, sICAM). All these mediators involved in inflammation, coagulation, endothelial activation, cell death and tissue repair were simultaneously measured by a single operator (MS) blinded to the clinical data. sST2 was measured with human magnetic Luminex screening assay (R&D, Bio-Techne, Lille, France), and the remaining sepsis mediators were measured with a multi-analyte Milliplex human cytokine kit (Millipore Corporation, Billerica, MA, USA). Sepsis mediators were analyzed using fluorescence intensities rather than concentrations, since conversion to concentration resulted in a loss of power [18].

### Statistical analysis

The data were analyzed using the IBM SPSS Statistics for Windows (version 19.0, IBM Corp Armonk, NY) and R 3.1.2 (The R Foundation for Statistical Computing, Vienna, Austria). Continuous data were expressed as medians [25th–75th centiles] unless otherwise specified and were compared using the nonparametric Kruskal–Wallis test followed by pairwise Mann–Whitney test. Correlations were tested using the nonparametric Spearman’s method. Categorical variables, expressed as percentages, were evaluated using the Chi-square test or Fisher’s exact test. The two aims of our study were achieved as follows. First, we tested the biological coherence of various sepsis mediators using hierarchical clustering; this method builds homogeneous clusters based on dissimilarities or distances between cases and proceeds iteratively to join the most similar cases. Second, we assessed the role of sepsis mediators on cardiac contractility, organ dysfunction and outcome by using bivariate correlations that were further summarized using focused principal component analysis (FPCA, “psy” package within the R environment) [19]. FPCA is a graphical representation similar to principal component analysis, but adapted to data with dependent/independent variables. We also explored potential interactions among sepsis mediators involved in septic myocardial dysfunction by assessing synergy factors as proposed by Cortina-Borja et al. [20]. Synergy factor is an easy-to-use and clear-to-interpret statistic to measure both the size and significance of binary interactions in complex diseases with all types of susceptibility factors, both risk and protective [21]. Synergy factor is calculated as the ratio of the observed odds ratio for both factors combined to the predicted odds ratio assuming independent effects of each factor. If synergy factor > 1 (< 1) with statistical significance, then there is a positive (negative) interaction between two risk factors. To calculate synergy factors, we partitioned the population for each tested mediator into two sets with higher versus lower concentrations based on the median value of fluorescence intensities [20]. All multiple comparisons (correlations and synergy factors) were corrected with Benjamini–Hochberg method to control the false discovery rate at the 0.05 level [22]. Significant univariate risk factors were used to adjust the association of sepsis mediators with ICU mortality using backward stepwise logistic regression analysis; to simplify their interpretation, sepsis mediator results were given for 1 standard deviation of the log-transformed fluorescence intensity of the cohort for this analysis [23]. Two-tailed *p* values < 0.05 were considered significant.

## Results

### Patient characteristics

Among 326 patients screened for septic shock during the study period, 252 were excluded because of one of the following reasons: chronic heart failure (*n* = 105), immunosuppression (*n* = 59), moribund state (*n* = 34), poor transthoracic echogenicity with contraindication to transesophageal route (*n* = 6), or sonographer or echocardiograph unavailability (*n* = 48). Thus, the present study comprises 74 patients (47 men and 27 women). The main source of infection was pulmonary (49%).

### Biological coherence of sepsis mediators

**Fig. 1:**
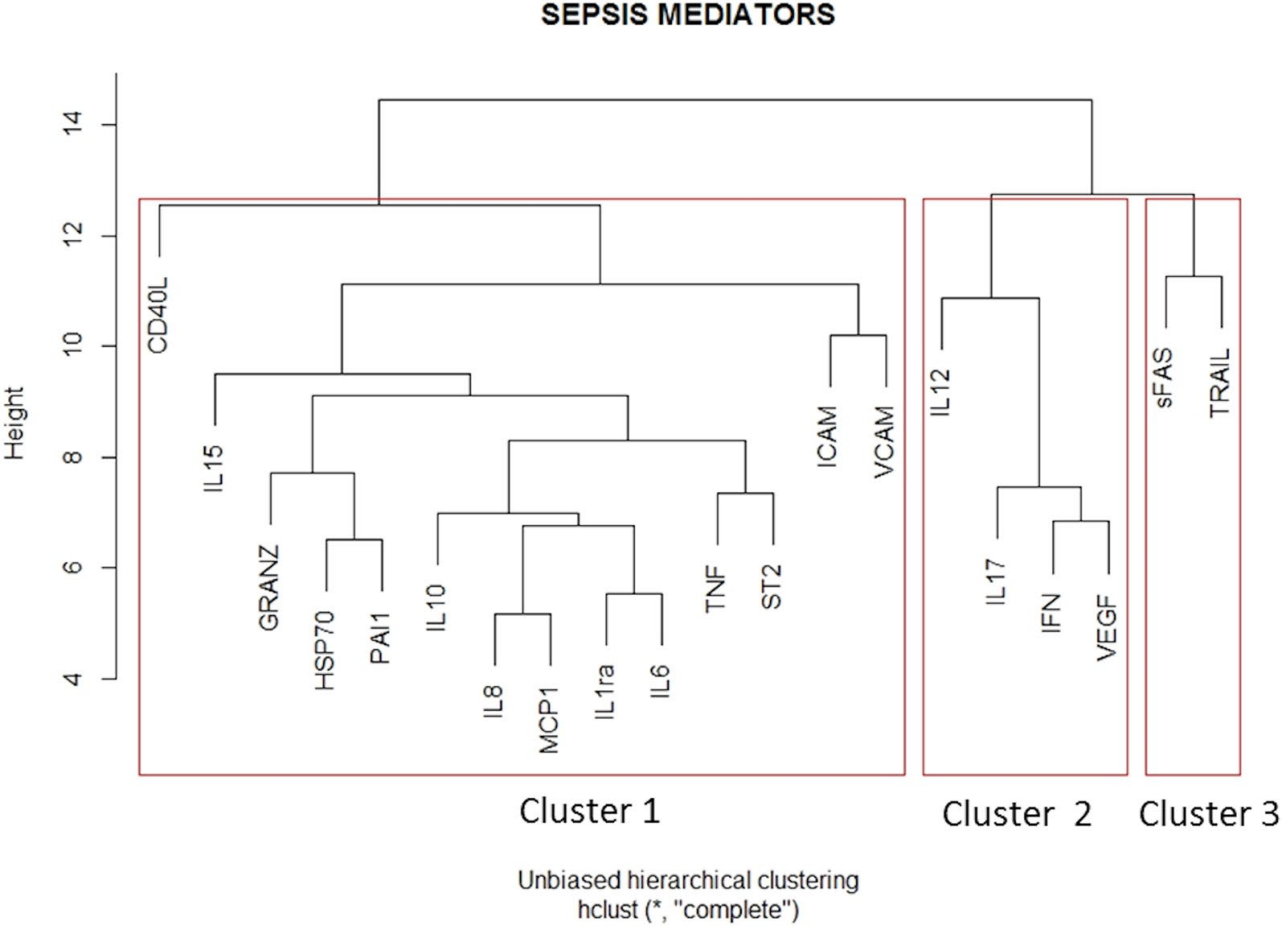
Hierarchical clustering of all sepsis mediators. The parameters were reordered using computerized hierarchical clustering with the corrplot package of R statistical environment. Hierarchical clustering is a statistical method for finding comparatively homogenous clusters of cases based on measured characteristics. The algorithm uses a set of dissimilarities or distances between cases when constructing the clusters and proceeds iteratively to join the most similar cases. Distances between clusters were recomputed by the Lance–Williams dissimilarity update formula according to the complete linkage method

IL-1α, IL-1β, IL-33 and sFAS ligand were excluded from analyses due to unreliable concentration ranges. An unsupervised computer-generated hierarchical clustering of the remaining 20 sepsis mediators was performed without consideration of clinical data. This analysis identified three main clusters suggesting coherent biological pathways (Fig. 1): The first cluster (CD40L, IL-8, MCP1, IL1-RA, IL-6, IL-15, TNF-α, IL-10, ICAM, sST2, VCAM, granzyme, HSP70 and PAI1) mainly involved innate immunity; the second cluster (IL-12, IL-17, IFN, VEGF) mainly involved adaptive immunity and repair; and the third cluster (sFAS, TRAIL) was related to cell death.

### Septic myocardial dysfunction

A total of 112 echocardiographies were performed during the first 2 days of septic shock in the 74 patients. LVEF was assessed using the Simpson method for 78 echocardiographies (70%) and visually estimated for the remainders. Septic myocardial dysfunction was diagnosed in a total of 24 patients (32%). Clinical characteristics and comorbidities were similar between patients with or without hypokinesia except for a higher SAPS II score at ICU admission in the former group; this group also exhibited more organ failures than the latter, but with similar mortality (see Additional file 1: Tables S1 and S2).

### Association of sepsis mediators with septic myocardial dysfunction

**Fig. 2:**
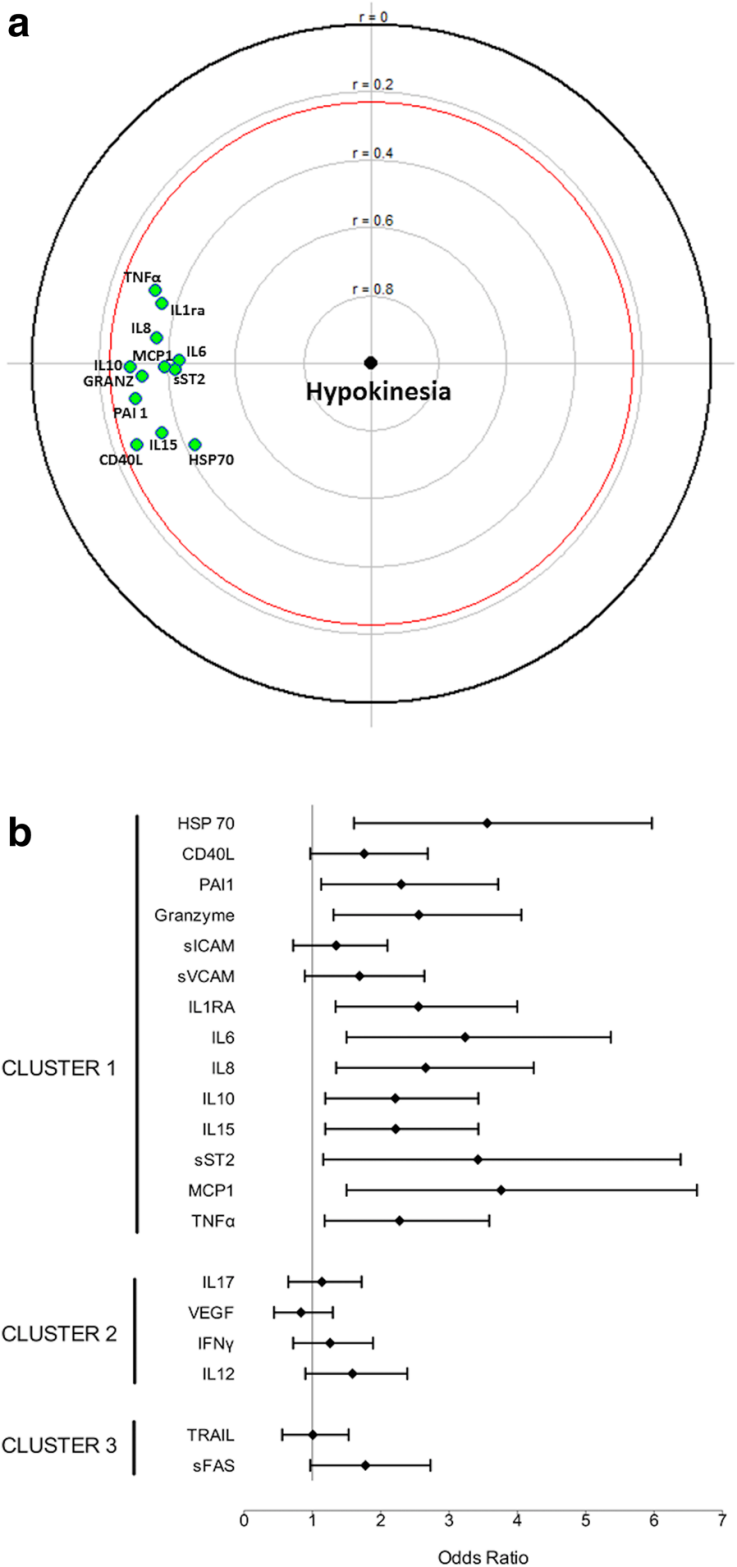
Forest plot for odds ratios (**a**) and focused principal component analysis (FPCA, **b**) for the association between sepsis mediators and septic myocardial dysfunction. FPCA is a simple graphical display of correlation structures focusing on a particular dependent variable. The display reflects primarily the correlations between the dependent variable and all other variables (covariates) and secondarily the correlations among the covariates. The dependent variable (septic myocardial dysfunction, SMD) is at the center of the diagram, and the distance of this point to a covariate faithfully represents their pairwise Spearman correlation coefficient (using ranked values of continuous variables). Green covariates are positively correlated with the dependent variable. For parsimony, only covariates significantly correlated with the dependent variable (with a *p* value < 0.05 and inside the red circle) are displayed. The diagram also shows relationships between covariates as follows: Correlated covariates are close (for positive correlations, allowing identification of clusters) or diametrically opposite vis-à-vis the origin (for negative correlations), whereas independent covariates make a right angle with the origin. See text for sepsis mediators’ abbreviations

**Fig. 3:**
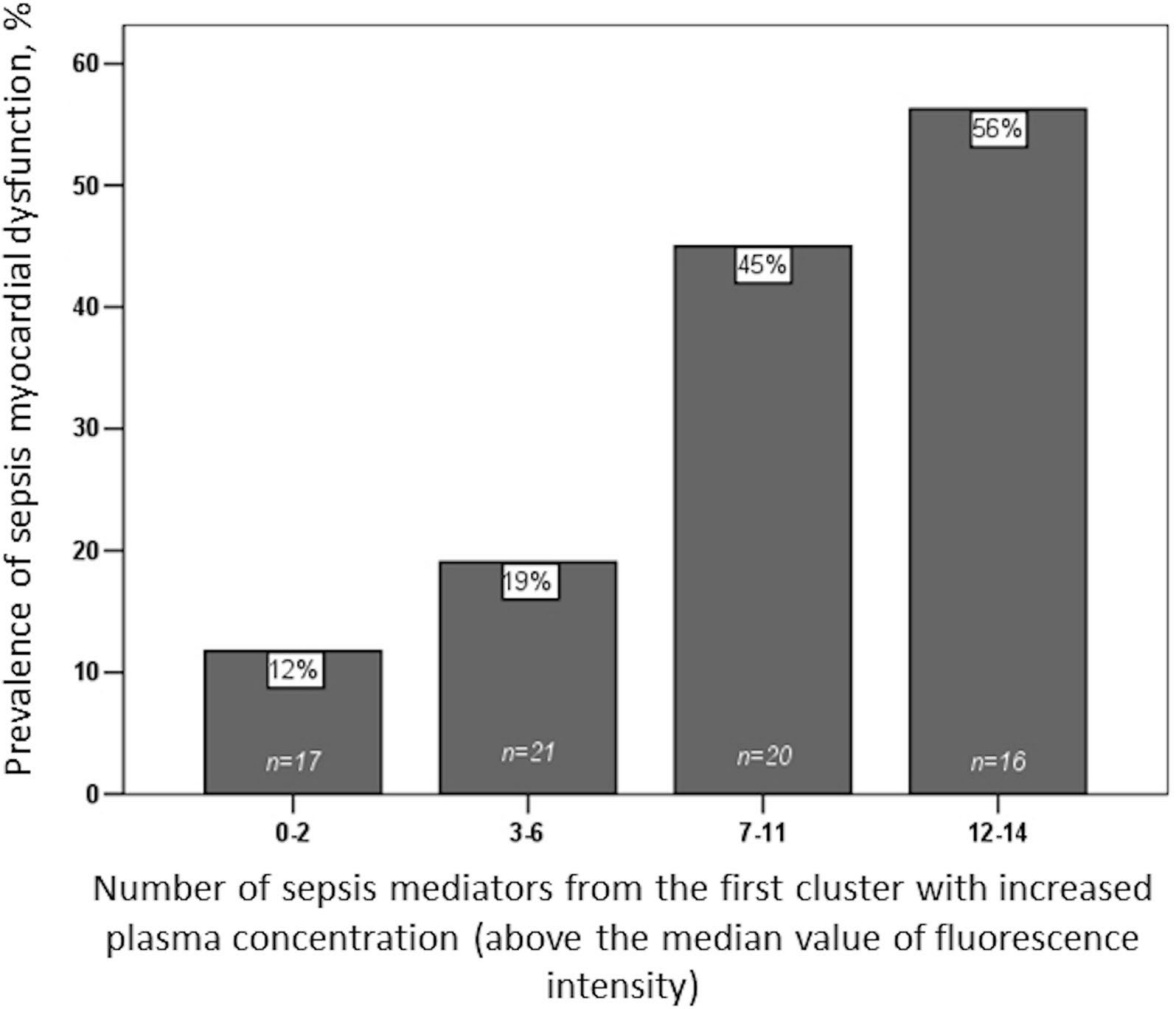
Prevalence of septic myocardial dysfunction according to the number of increased sepsis mediators (above the median value) from the first cluster

All inflammatory markers significantly associated with septic myocardial dysfunction (CD40L, IL-8, MCP1, IL1-RA, IL-6, IL-15, TNF-α, sST2, granzyme and HSP70) belonged to the first cluster (Fig. 2a, b). The prevalence of septic myocardial dysfunction increased linearly with the number of augmented sepsis mediators (above the median value of fluorescence intensity), suggesting an additive effect on the risk of myocardial dysfunction (Fig. 3). The number of augmented sepsis mediators (above the median value of fluorescence intensity) from the first cluster remained associated with septic myocardial depression even after adjusting on patient’s severity (using SAPS II score at ICU admission): adjusted odds ratio of 1.25, 95% CI 1.08–1.45, *p* = 0.004. Ninety-one pairs of mediators of the first cluster were assessed for synergy factor analysis: Almost all of them (89 pairs, 98%) showed no significant interaction, also suggesting an additive effect (see Additional file 1: Table S3).

### Association of sepsis mediators with organ failures and outcome

**Fig. 4:**
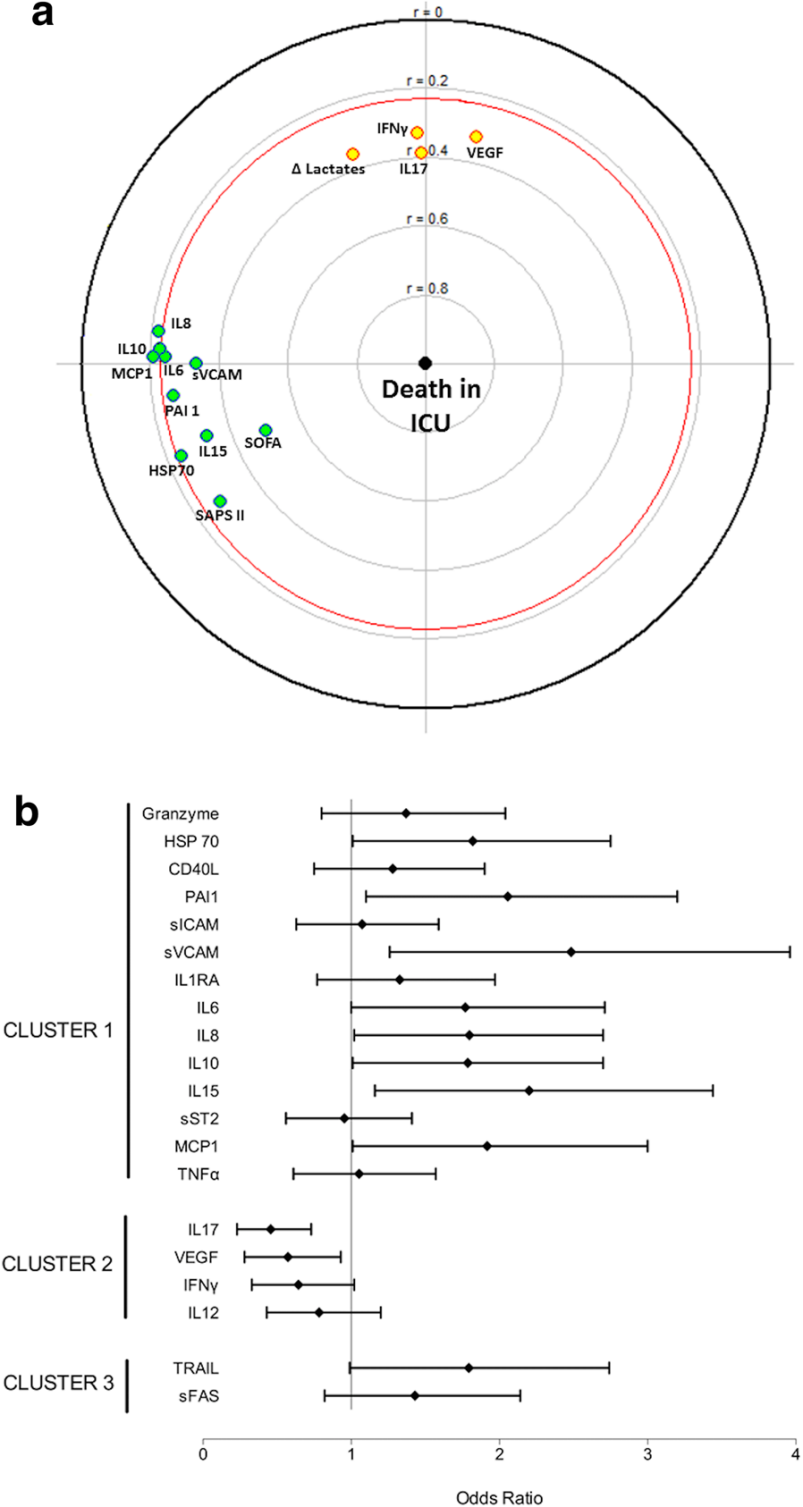
Forest plot for odds ratios (**a**) and focused principal component analysis (FPCA, **b**) for the association between sepsis mediators and intensive care unit mortality. **b** Correlation of ICU mortality (dependent variable at the center) with sepsis mediators at day 1, patient’s severity (as assessed by SAPS II score), organ dysfunction (as assessed by SOFA score at septic shock onset) and lactate clearance (relative difference between lactate concentration at septic shock onset and after 24 h of resuscitation) as a surrogate of early sepsis resolution. See Fig. 2 legend for details on FPCA. Variables positively and negatively correlated with ICU mortality are in green and yellow, respectively. See text for sepsis mediators’ abbreviations

Many mediators from the first cluster were associated with patient’s severity (as assessed by SAPS II score), organ failures (as assessed by SOFA score) and death (Fig. 4a, b). On the contrary, most mediators from the second cluster (IL-17, IFN-γ and VEGF) were associated with a surrogate of early sepsis resolution (lactate clearance at 24 h) and ICU survival (Fig. 4a, b). The association of IL-17 (chosen as a surrogate of the second cluster) with ICU survival persisted (adjusted odds ratio of 0.33, 95% CI 0.17–0.66, *p* < 0.001) after adjustment on patient’s severity (using SAPS II score at ICU admission), organ failures (using SOFA score at septic shock onset) and IL-15 concentration (chosen as a surrogate of the first cluster to reflect innate immunity) (see Additional file 1: Table S4).

## Discussion

Unlike most studies reported in the literature, we have comprehensively explored plasma mediators in human septic shock with a multiplex assay and specific statistical tools. We identified three clusters suggestive of meaningful biological pathways. One cluster with innate inflammatory cytokines was associated with myocardial and other organ dysfunction, whereas another cluster involving the adaptive immunity was associated with surrogates of early sepsis resolution and in ICU survival.

### Myocardial depressant factor

The first cluster included innate inflammatory cytokines such as IL-6, IL1-RA, TNF-α, MCP1, IL-8 and sST2. The majority of these cytokines have been previously individually associated either with septic myocardial dysfunction or with mortality during septic shock. Parrillo et al. [3] were the first in 1985 to suggest the presence of circulating myocardial depressant substance(s) in the sera of septic shock patients. Indeed, sera obtained in the acute phase of septic shock patients with septic myocardial dysfunction decreased in vitro myocardial cell shortening. Moreover, sera from the same patients before shock or after recovery and sera from patients with other cardiac disease or from critically ill non-septic patients induced no significant changes. Kumar showed that TNF-α and IL-1β induced cardiac myocyte depression in vitro [7]. Immunoabsorption of both TNF-α and IL-1β abrogated in vitro myocardial depressant activity of sera from humans with septic shock. Trials conducted in the 1990s with a monoclonal TNF-α antibody during septic shock revealed a transient improvement in cardiovascular parameters [24], but failed to alter the mortality rate of patients [25]. Using gene microarray analysis, Pathan et al. [26] suggested that IL-6 was a mediator of myocardial depression in children with meningococcal septic shock. Other candidates for myocardial depressant factor have also been recently suggested. Our study is the first to show a correlation between septic myocardial dysfunction and sST2 (an interleukin-1 receptor family member which is markedly induced by mechanical strain in cardiac myocytes) [27]. Most mediators of the first cluster had an additive effect on septic myocardial dysfunction. Our study suggests that a cluster of mediators involved in innate immunity rather than an individual factor might influence the onset of septic myocardial dysfunction. At the cellular level, this dysfunction may be mediated through the alteration of other downstream cellular mediators (e.g., sphingomyelinase, nitric oxide or phospholipase A2-dependent signaling).

Our results contradict some previous reports. Bouhemad et al. found no correlation between three cytokines (TNF-α, IL-8 and IL-10) and myocardial systolic dysfunction [28]. Landesberg et al. also failed to show a significant correlation between inflammatory cytokines (IL-1β, IL-6, IL-8, IL-10, IL-18, TNF-α and MCP1) and systolic dysfunction [9]. The above-mentioned studies included a limited number of patients with systolic myocardial dysfunction (11 and 13 patients, respectively). In addition, plasma cytokines were collected within 2 days after the diagnosis of sepsis. This delay may have underestimated the initial peak concentrations [29]. Cardiomyocyte injury or death, maybe theoretically induced by toxins, complements or DAMP during septic shock. However, factors involved in apoptosis (sFAS, TRAIL) and belonging to cluster 3 were not associated with septic myocardial dysfunction in our study. These results are in line with autopsy studies, which did not show evidence of irreversible acute injury or cell death in deceased patients with septic myocardial dysfunction [30].

### Organ failures and outcome

Our finding that cytokines from the first cluster were associated with morbidity and mortality is in accordance with previously published studies [9, 31–34]. Interestingly, the second cluster involving IL-17/IFN pathway and VEGF correlated tightly with a surrogate of early sepsis resolution (lactate clearance) and was significantly associated with ICU survival. CD4^+^ T helper (Th) cells play a central role in the adaptive immune response by stimulating B cells and cytotoxic T cells and by releasing different types of cytokines in tissues to mediate protection against a wide range of pathogenic microorganisms. IFN-γ-producing Th1 cells have typically been associated with immune response to viruses and intracellular bacteria, and IL-17-producing Th17 play a role in immunity against fungi and extracellular bacteria. Impaired Th17 responses diminish bacterial clearance and increase epithelial vulnerability [35]. Our results are in line with previous data, suggesting that Th17 differentiation impacts mortality during severe sepsis [36]. Increased levels of IL-17 and IFN may reflect an effective inflammatory response to infection with enhanced bacterial clearance [37, 38]. Moreover, IL-17 can upregulate VEGF [39] to restore mucosal endothelial/epithelial function and stimulate healing [40]. Our study suggests that it is rather the initial balance between mediators of “innate immunity” (the first cluster) and those of “adaptive immunity and repair” (the second cluster) than a two-stage process that determines organ failure and outcome during septic shock. Early cytokine profiling may be a molecular tool helpful to identify different patient populations and a useful instrument for future clinical trials targeting the immune system in patients with septic shock.

### Strengths and limitations

The strengths of our study include its prospective design, the severity of the patients selected, the multiplex assay of sepsis mediators and the comprehensive analysis with control for false discovery rate. Limitations of our study include its monocentric design, the relatively small number of patients and the lack of a validation cohort. Our findings need an independent validation in a similarly well-phenotyped cohort. Although patients with known chronic heart failure were excluded from the study, we could not completely exclude occult chronic heart failure in some cases. Among the 24 patients with septic myocardial dysfunction in our study, 19 had a preserved LVEF on a baseline echocardiography performed before ICU admission or a follow-up echocardiography performed 1 week after septic shock. Only five patients had no baseline echocardiography and no follow-up echocardiography (because they died within the first 2 day of septic shock); all these five patients had an elevated troponin concentration, suggesting sepsis myocardial depression was likely [41] and occult chronic heart failure was unlikely. Despite these limitations, our study shows that measuring some sepsis mediators may be helpful to depict robust patient profiles.

## Conclusion

In this preliminary study, a comprehensive analysis of sepsis mediators revealed meaningful biological pathways during human septic shock. One cluster involved in innate immunity was associated with septic myocardial dysfunction and organ failures. In contrast, another cluster involving IL-17/IFN pathway and VEGF correlated with early sepsis resolution and was associated with ICU survival. Our findings may enrich the design of future clinical trials targeting the cardiovascular and immune systems in patients with septic shock.

## Additional file

**Additional file 1. Table S1.** Characteristics of patients with septic shock according to septic myocardial dysfunction (n=74). **Table S2.** Characteristics of patients with septic shock according to outcome (n=74). **Table S3.** Matrix of synergy factors exploring interactions between sepsis mediators of the first cluster as susceptibility factors of septic myocardial dysfunction. **Table S4.** Multivariable analysis of factors associated with death in intensive care unit during septic shock by logistic regression.

## Data Availability

The datasets generated during and/or analyzed during the current study are not publicly available as consent for publication of raw data was not obtained from study participants, but are available from the corresponding author on reasonable request.
