# Supplementary material for: A multiplex analysis of sepsis mediators during human septic shock: a preliminary study on myocardial depression and organ failures
Source: Ann Intensive Care. 2019 Jun 4;9:64. doi: 10.1186/s13613-019-0538-3 (PMC6548788; doi:10.1186/s13613-019-0538-3)
Supplement: Supplementary file 1 — Additional file 1. Table S1. Characteristics of patients with septic shock according to septic myocardial dysfunction (n=74). Table S2. Characteristics of patients with septic shock according to outcome (n=74). Table S3. Matrix of synergy factors exploring interactions between sepsis mediators of the first cluster as susceptibility factors of septic myocardial dysfunction. Table S4. Multivariable analysis of factors associated with death in intensive care unit during septic shock by logistic regression. [file 13613_2019_538_MOESM1_ESM.docx]

**Table S1. Characteristics of patients with septic shock according to septic myocardial dysfunction (n=74).**

|  | **No hypokinesia**  **(n=50)** | **Hypokinesia**  **(n=24)** | ***P* value** |
| --- | --- | --- | --- |
| **Clinical characteristics and comorbidities** |  |  |  |
| Age (years) | 65 [48-78] | 68 [57-80] | 0.33 |
| Male gender, n (%) | 30 (60%) | 17 (71%) | 0.37 |
| Chronic Obstructive Pulmonary Disease | 2 (%) | 0 | >0.99 |
| Chronic kidney disease requiring long-term dialysis | 2 (4%) | 1 (4%) | >0.99 |
| Liver cirrhosis | 1 (2%) | 0 | >0.99 |
| Mc Cabe and Jackson class  0  1  2 | 33 (66%)  16 32%)  1 (2%) | 19 (79%)  5 (21%)  0 (0%) | 0.45 |
| SAPS II at ICU admission | 49 [35-63] | 58 [48-86] | 0.02 |
| Source of infection — no. (%)  Lung  Abdomen  Urinary tract  Others | 28 (56%)  7 (14%)  10 (20%)  5 (10%) | 8 (33%)  6 (25%)  5 (21%)  5 (21%) | 0.23 |
| Bacteraemia | 25 (50%) | 14 (58%) | 0.50 |
| Gram negative bacilli | 32 (64%) | 12 (50%) | 0.25 |
| Surgery | 14 (29%) | 7 (29%) | >0.99 |
| Appropriateness of empirical antimicrobial therapy | 46 (92%) | 23 (96%) | >0.99 |
| **Organ failures**# |  |  |  |
| Sequential Organ Failure Assessment score | 10 [8-12] | 12 [9-15] | 0.04 |
| Arterial lactates (mmol/L) | 2.3 [1.2-4.1] | 3.4 [2.4-6.5] | 0.03 |
| PaO_2_/FiO_2_ ratio (mmHg) | 191 [104-256] | 148 [99-295] | 0.66 |
| Serum creatinine (mmol/L) | 142 [75-234] | 246 [141-314] | 0.01 |
| Dose of norepinephrine (µg/kg/min) | 0.65 [0.30-1.28] | 1.12 [0.29-2.02] | 0.35 |
| **Outcome** |  |  |  |
| Dobutamine during ICU | 0 | 13 (54%) |  |
| Epinephrine during ICU | 0 | 5 (21%) |  |
| Mechanical ventilation | 43 (86%) | 21 (88%) | >0.99 |
| Moderate-to-severe ARDS | 21 (42 %) | 9 (38%) | 0.80 |
| Dialysis for acute renal failure | 10 (20%) | 7 (29%) | 0.38 |
| LOS in ICU, all patients (days) | 11 [5-28] | 6 [2-12] | 0.04 |
| LOS in ICU, survivors only (days) | 13 [6-24] | 10 [6-24] | 0.82 |
| Death in ICU | 21 (42%) | 9 (38%) | 0.71 |
| Death in hospital | 22 (44%) | 9 (38%) | 0.60 |

Abbreviations: SAPS= Simplified Acute Physiology Score, ARDS= Acute Respiratory Distress Syndrome; ICU= Intensive Care Unit, LOS= length of stay, #available data were averaged over the first 48 hours of septic shock.

**Table S2. Characteristics of patients with septic shock according to outcome (n=74).**

|  | **ICU survival**  **(n=44)** | **ICU death**  **(n=30)** | ***P* value** |
| --- | --- | --- | --- |
| **Clinical characteristics and comorbidities** |  |  |  |
| Age (years) | 64 [51-76] | 69 [52-79] | 0.25 |
| Male gender, n (%) | 27 (61%) | 20 (67%) | 0.81 |
| Chronic Obstructive Pulmonary Disease | 1 (2%) | 1 (3%) | >0.99 |
| Chronic kidney disease requiring long-term dialysis | 2 (5%) | 1 (3%) | >0.99 |
| Mc Cabe and Jackson class  0  1  2 | 13 (75%)  11 (25%)  0 | 19 (63%)  10 (33%)  1 (3%) | 0.33 |
| SAPS II at ICU admission | 50 [36-61] | 62 [47-87] | 0.02 |
| Community acquired infection  Health care associated infection  Nosocomial infection | 27 (61%)  5 (11%)  12 (27%) | 15 (50%)  5 (17%)  10 (33%) | 0.61 |
| Source of infection — no. (%)  Lung  Others | 17 (39%)  27 (61%) | 19 (63%)  11 (37%) | 0.037 |
| Bacteraemia | 26 (59%) | 13 (43%) | 0.18 |
| Gram negative bacilli | 26 (59%) | 18 (60%) | >0.99 |
| Surgery | 16 (37%) | 5 (17%) | 0.06 |
| Appropriateness of empirical antimicrobial therapy | 41 (93%) | 28 (93%) | >0.99 |
| **Organ failure at day-1** |  |  |  |
| Maximal dose of norepinephrine at day1 (µg.kg^-1^.min^-1^) | 0.67 [0.27-1.22] | 0.83 [0.45-2.6] | 0.14 |
| SOFA score at day-1 | 9 [8-11] | 13 [11-15] | <0.001 |
| Mechanical ventilation at day-1 | 35 (80%) | 26 (87%) | 0.43 |
| Moderate-to-severe ARDS at day-1 | 14 (32%) | 14 (47%) | 0.20 |
| Arterial lactate at day-1 (mmol/L) | 2.6 [1.5-3.9] | 3.2 [1.3-7.1] | 0.30 |

Data are median [1^st^ quartile-3^rd^ quartile] or number (percentage); Abbreviations: SAPS= Simplified Acute Physiology Score; ARDS= Acute Respiratory Distress Syndrome; ICU= intensive care unit, SOFA= Sequential Organ Failure Assessment;

**Table S3. Matrix of synergy factors exploring interactions between sepsis mediators of the first cluster as susceptibility factors of septic myocardial dysfunction.**

| **Synergy factors** | **HSP70** | **sICAM** | **sVCAM** | **IL1RA** | **IL6** | **IL8** | **IL10** | **IL15** | **sST2** | **MCP1** | **TNFα** | **PAI1** | **sCD40L** |
| --- | --- | --- | --- | --- | --- | --- | --- | --- | --- | --- | --- | --- | --- |
| **Granzyme** | 0.40 | 1.33 | 0.26 | 2.03 | 11.2 | 3.33 | 8.8 | 96.0* | 1.6 | 11.9 | 0.31 | 2.0 | 9.5 |
| **HSP70** |  | 4.6 | 1.3 | 0.03 | 0.20 | 0.23 | 0.85 | 2.2 | 0.4 | 0.56 | 0.46 | 0.16 | 0.73 |
| **sICAM** |  |  | 2.7 | 0.29 | 0.98 | 0.54 | 0.76 | 3.3 | 0.68 | 0.72 | 1.4 | 1.3 | 0.30 |
| **sVCAM** |  |  |  | 0.13 | 0.14 | 0.23 | 0.34 | 1.2 | 0.05 | 0.37 | 0.27 | 0.27 | 2.6 |
| **IL1RA** |  |  |  |  | 0.97 | 0.44 | 1.6 | 2.03 | 1.16 | 2.7 | 0.35 | 0.51 | 0.62 |
| **IL6** |  |  |  |  |  | 0.69 | - | 3.6 | 0.83 | 7.2 | 0.37 | 3.6 | 0.19 |
| **IL8** |  |  |  |  |  |  | 1.7 | 12.9 | - | - | 0.34 | 0.22 | 1.01 |
| **IL10** |  |  |  |  |  |  |  | 18.4 | 0.89 | 5.97 | 0.42 | 8.81 | 4.85 |
| **IL15** |  |  |  |  |  |  |  |  | 7.71 | 49.5* | 2.95 | 9.37 | 2.51 |
| **sST2** |  |  |  |  |  |  |  |  |  | 4.46 | 0.31 | 0.99 | 0.82 |
| **MCP1** |  |  |  |  |  |  |  |  |  |  | 0.78 | 1.35 | 3.47 |
| **TNFα** |  |  |  |  |  |  |  |  |  |  |  | 0.20 | 1.77 |
| **PAI1** |  |  |  |  |  |  |  |  |  |  |  |  | 2.51 |
| **sCD40L** |  |  |  |  |  |  |  |  |  |  |  |  |  |

*p<0.05 after Benjamini-Hochberg correction

**Table S4. Multivariable analysis of factors associated with death in intensive care unit during septic shock by logistic regression.**

|  | **Odds Ratio (95% Confidence Interval), p value** | |
| --- | --- | --- |
| **Predictor** | **Univariate** | **Adjusted (multivariable)** |
| SAPS II score at admission (per point) | 1.04 (1.02-1.05), p<0.001 | NS |
| SOFA score on the first day of septic shock (per point) | 1.3 (1.16-1.44), p<0.001 | 1.7 (1.3 - 2.2), p<0.001 |
| IL17 (per SD of the log transformed fluorescence intensity) | 0.41 (0.23-0.73),p=0.003 | 0.33 (0.17 – 0.66), p=0.001 |
| IL15 (per SD of the log transformed fluorescence intensity) | 2.0 (1.2-3.4), p=0.012 | NS |

The variables included in the analysis were: SAPS II at admission, SOFA score on the first day of septic shock, IL17 and IL15;

Abbreviations: NS: not significant; SOFA= Sequential Organ Failure Assessment; SAPS= simplified acute physiology score, SD=standard deviation.
